# Mobile Acoustic Wave Platform Deployment in the Amazon River: Impact of the Water Sample on the Love Wave Sensor Response

**DOI:** 10.3390/s20010072

**Published:** 2019-12-21

**Authors:** Ollivier Tamarin, Maxence Rube, Jean Luc Lachaud, Vincent Raimbault, Dominique Rebière, Corinne Dejous

**Affiliations:** 1 Université de Guyane, UMR 228 Espace-Dev, F-97300 Cayenne, France; maxence.rube@etu.univ-guyane.fr; 2 Université de Bordeaux, CNRS, Bordeaux INP, IMS, UMR 5218, F-33400 33400 Talence, France; jean-luc.lachaud@ims-bordeaux.fr (J.L.L.); dominique.rebiere@recherche.gouv.fr (D.R.); corinne.dejous@ims-bordeaux.fr (C.D.); 3 LAAS, CNRS UPR 8001, F-31031, 31400 Toulouse, France; vraimbau@laas.fr

**Keywords:** love wave acoustic sensor, liquid sensing, in field operation, microfluidic, portable electronic platform

## Abstract

This paper presents an experimental platform allowing in situ measurement in an aqueous medium using an acoustic Love wave sensor. The aim of this platform, which includes the sensor, a test cell for electrical connections, a microfluidic chip, and a readout electronic circuit, is to realize a first estimation of water quality without transportation of water samples from the field to the laboratory as a medium-term objective. In the first step, to validate the ability of such a platform to operate in the field and in Amazonian water, an isolated and difficult-to-access location, namely, the floodplain Logo Do Curuaï in the Brazilian Amazon, was chosen. The ability of such a platform to be transported, installed on site, and used is discussed in terms of user friendliness and versatility. The response of the Love wave sensor to in situ water samples is estimated according to the physical parameters of Amazonian water. Finally, the very good quality of the acoustic response is established, potential further improvements are discussed, and the paper is concluded.

## 1. Introduction

The Amazonian area, which is localized in nine countries of South America (Brazil, Bolivia, Peru, Ecuador, Colombia, Venezuela, Guyana, Suriname, and French Guiana), is experiencing a period of fast development based on political strategies whose objectives are to exploit the resources of its soil, underground, and rivers. As examples, human activities involving mining, agriculture, energy, and transport infrastructure are spread over increasingly large Amazonian virgin territories. Such activities cause some problematic issues and environmental changes that could irreversibly interfere with, or even completely disrupt, the fauna, the flora, and human health in these areas [1,2]. Conversely, the residents need long-term resources and economic activities. From this point of view, technological tools could help to monitor, control, and prevent pollution and disasters before they occur.

Environmental surveys of Amazonian areas, especially in aqueous media such as rivers, lakes, and artificial water reservoirs, are usually performed using classical methods that we can present along two principal axes: remote sensing and biochemical analysis of in situ collected samples. Remote sensing offers the advantage of being easy to implement with the appropriate software to analyze a large surface area using satellite images [3,4]. Nevertheless, this method is not appropriate for the estimation of biochemical compounds, especially in the water column, except in some specific cases [5].

The other method is based on the collection of in situ samples with conditioning methods that require not only the transport of the samples from the field to analytical laboratories but also the analysis of these same samples by highly qualified personnel using analytical chemistry tools that are sometimes very expensive, such as gas chromatographs, mass spectrometers, and electrophoretic stations.

Drawbacks include the biochemical degradation of the samples during their collection and transportation [6]. Moreover, this method does not allow an exhaustive analysis of the biochemical compounds in the water column over a large area of investigation. Indeed, the logistics of deployment and the overall cost, including consumables and the workforce, result in a very low frequency of in-field sample collection and necessitate complex work to evaluate a large number of samples that nevertheless cannot be efficiently treated in terms of time compared to the surfaces to be studied. This drawback is not compatible with the need for water quality data over a large area [7].

An intermediate approach, allowing the coverage of a high surface area while analyzing biochemical compounds in the water column, is to use in situ biochemical sensors that are easily deployable. If one sensor can provide information about the presence of a target compound, certain properties are important for covering a large area with a minimum cost, such as good sensitivity, easy communicating, low cost, user friendliness, and low power consumption or even passive power operation. These properties leverage autonomous sensor networks to cover a large area and/or to allow the use of nonexpert people to realize rapid in situ testing of water quality.

As a response to this issue, we propose to use acoustic transduction based on a surface acoustic wave (SAW) filter with the ultimate goal to realize biochemical detection in the field. SAW sensors are a good candidate to supply all the needed properties for such a sensor [8,9]. As they are used as filters [8], they can be mass produced at a low cost in a very small size, and they are well adapted for integration in a communication system based on the Internet of Things concept [10].

This choice is based first on the fact that acoustic wave transduction could be complementary to or even better than optical transduction in Amazonian rivers, to avoid light flux optical absorption, which can occur in turbid water [11]. Second, SAW devices present good properties as sensors in both gas and aqueous media, such as good sensitivity [9,12], easy functionalization due to their sensitive surface area [13,14], and good accessibility for integration of microfluidic circuits in aqueous media [7,15,16,17].

As much work has already been performed involving biological and chemical detection of molecules using Love wave devices in aqueous media, in this first step we only focus on a sensor without functionalization. The aim of our work is to validate the Love wave sensor to operate in real Amazonian water using an experimental setup that is transportable and not expensive and directly usable in the field. The paper is organized in three main parts. We first present the acoustic wave transducers used, the readout electronic and microfluidic setup used, and the capacity for transport in difficult-to-access environments. The second part presents the method for the detection tests and the localization of the collected samples in the Lago do Curuaï, which is the site in which these experimental tests were carried out. Finally, prior to the conclusion of the paper, the third part presents the results and the discussion about the ability of this system to operate in Amazonian waters. Improvements, as well as perspectives for biochemical species detection in the near future, are also presented.

## 2. Portable Experimental Setup Based on Love Wave Transduction

### 2.1. Constitutive Elements of the Experimental Platform

#### 2.1.1. Love Wave Transducer

The experimental platform we used is based on a SAW sensor and, more specifically, on Love wave propagation. As described before, this choice is based on the fact that acoustic transduction could be complementary to the optical transduction; in addition, the Love wave device, which is based on a shear horizontal (SH) wave propagating in a thin layer deposited on a piezoelectric substrate, is able to operate in aqueous media without excessive acoustic energy loss [9,17]. Thus, the sensing mechanism is essentially based on the mass effect (amount of mass deposited on the surface of the sensor) but also on the overall mechanical and electrical aqueous media properties, including in particular the density, viscosity, thickness [17,18,19], and electrical conductivity [20,21].

**Figure 1:**
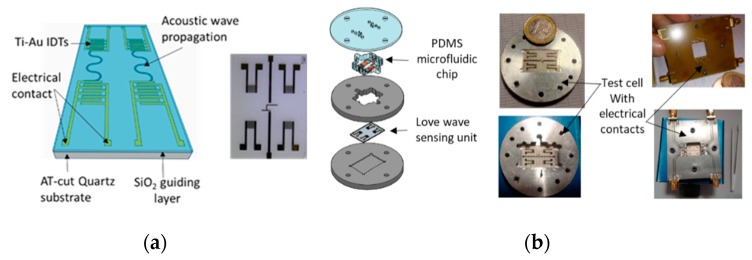
Dual Love wave sensor description (**a**) and photograph (middle), with an experimental test cell dedicated for aqueous samples (**b**).

The device we used is a Love wave delay line based on a 500 µm thick AT-cut quartz piezoelectric substrate with a 6.4 µm SiO_2_ guiding layer, as presented in Figure 1a. The detailed geometry was already described in references [15,22,23]. The operating principle of the Love wave sensor is based on the generation of an acoustic wave by the piezoelectricity effect. Indeed, by applying an electrical signal on the electrical contact of the interdigitated electrodes (IDTs), a mechanical deformation is generated and propagates along the acoustic path between the IDTs (see Figure 1a). The sensing mechanism is based on the perturbation of the propagation parameters of the acoustic wave by a target compound or an aqueous medium, which modifies, among other parameters, the wave amplitude and velocity of the Love wave. The characterization of such a device in Amazonian water is then an important step to validate the ability of the acoustic wave to propagate without excessive attenuation of the wave amplitude, which enables otherwise-impossible biodetection tests by using our portable experimental platform.

The device we use is composed of a dual Love wave transducer to realize differential measurements with a sensing line and a reference line. For our experimental test, we used three different dual sensors that were fabricated with the same fabrication process and at the same time.

#### 2.1.2. Love Wave Test Cell

The Love wave sensor unit, which is the key element of the platform, is inserted in an experimental setup composed of three main parts, namely, a sensing unit with a Love wave sensor and a test cell, allowing electrical connections to ensure of the sensor. For application in aqueous media, a microfluidic chip is used for the localization of the aqueous sample on the surface of the sensor without touching the electrical contacts or the electronic readout circuit.

As presented in Figure 1 on the b side, the test cell and the microfluidic chip are designed to be easily usable. Indeed, all the components of the cell, including the microfluidic chip and the sensor can be assembled manually and are sealed by screws that hold the assembly together.

#### 2.1.3. Microfluidic Chip

The microfluidic chip is fabricated in Polydimethylsiloxane (PDMS) by using a SU8 negative mold with the appropriate dimensions, including not only the location of the aqueous sample on the sensitive surface of the sensor but also two orifices with a microfluidic inlet and outlet for the aqueous samples (see Figure 1b). The fabrication and the initial tests of this microfluidic chip are described in references [24,25]. According to the dual Love wave transducers used, two separate chambers are designed to allow the addition, removing, and localization of two aqueous samples of 200 µl each, independently, without contacts on any delay line. The strategy of localization of the water sample only on the SiO_2_ sensitive surface of the sensor, and not facing the IDTs, prevent high insertion losses due to the electromagnetic feedtrough in the presence of a liquid.

#### 2.1.4. Electronic Interrogation Module

**Figure 2:**
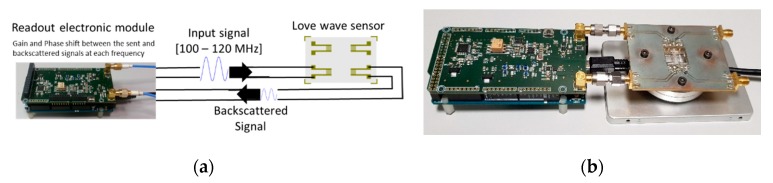
Readout electronic circuit operating under the Love wave sensor principle (**a**) and photograph of the device (**b**).

With the aim to make the device as inexpensive as possible and as simple as possible to use, we have realized an electronic module allowing the interrogation of the sensor for our platform. The operating principle of this module is based on the transmission analysis (S_21_) of a device under test (in our case, the Love wave sensor) by a Vector Network Analyzer (VNA). An electronic board has been designed that is composed of two main circuits: a direct digital synthesizer (AD 9954), which can send a sinusoidal electronic signal to the sensor with a frequency range between 100 MHz and 120 MHz, and an I/Q demodulator (AD 8302), which allows comparison of the input and output signals of the sensor, as shown in Figure 2 based on an initial design in reference [26]. This electronic board is controlled by an Arduino Due microcontroller (Arduino, Scarmagno, Italy) that allows not only monitoring and querying the sensor in the frequency range of the electronic board but also extracting the response of the sensor. The entire electronic module (card and microcontroller) is connected to a computer via a USB link allowing the powering and monitoring of the electronic circuit. Thus, by using customized Open source software, the calibration, supervision, operation, and storage of the measured data are implemented. We first measure the S_21_ parameters in terms of the phase and gain of the sensor in the frequency domain and then choose a fixed frequency and periodically monitor the response of the sensor according to the external disturbances on its surface. This second operating mode, also called “zero spanning”, measures the temporal variation in terms of the phase and insertion losses of the sensor.

### 2.2. Experimental Portable Test Platform for In-field Operations

#### 2.2.1. Portability and Deployability of the Platform

**Figure 3:**
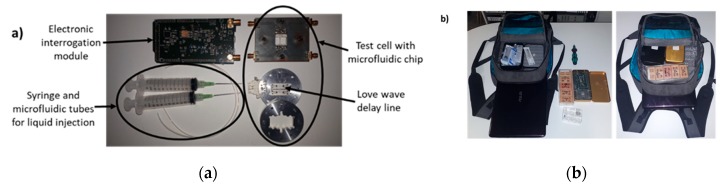
Love wave acoustic platform in the “transportable” configuration: (**a**) disassembled platform, (**b**) portable platform stored in a backpack.

Two main properties are important for the Love wave acoustic mobile platform: (i) an easily mountable setup that can be assembled and disassembled in a reasonable time with a minimum set of tools that are easy to obtain anywhere, and (ii) a platform that can be simply transported in the cabin of a plane, in a boat, in a car, or even by walking. In Figure 3a, the Love wave platform is presented in its disassembled configuration.

The Love wave delay line inserted inside the test cell with the microfluidic test cell is mounted in the field using four screws and a torque screwdriver to control the pressure exerted to hold the microfluidic chip on the sensor surface. This control is essential to not generate too much acoustic loss due to the absorption of the wave by the PDMS because of its presence in part of the acoustic path of the Love wave sensor.

Figure 3b presents the Love wave platform as configured for travel by using all the usual means of transport. Additional tools and equipment can also be stored in the backpack, such as a syringe, microfluidic tubes, and several Love wave sensors for operation (in this experiment on the Lago do Curuaï, we brought six sensors and used three), a home-made calibration “SOLT” kit, and a laptop to control the electronic readout interrogation module and store the measured data. For our experimental tests, the mobile platform was deployed completely (involving assembly of the Love platform, deployment of the computer and the control software of the platform, and calibration operations) in less than two hours by one operator.

#### 2.2.2. Additional Equipment for the In-field Operations

The aim of these experiments is to estimate the ability of the acoustic Love wave platform prototype to operate in Amazonian waters. To validate the results obtained, two additional instrumentation tools were used.

The first one is the Anritsu MS2028B handheld VNA (Anritsu, Atsugi, Japan) with an operating frequency between 5 kHz and 6 GHz. This tool was used to verify and compare the response of the readout electronic module to the VNA response while interrogating the Love wave sensor inserted in its test cell. These comparisons allow us to verify the behavior of the sensor to determine in the case of an incomprehensible response whether the issue is with the Love wave delay line or the electronic module. In addition, the performance of the electronic module can also be estimated. Note that during these experiments, no problem from the electronic module or the Love wave sensor was identified.

The second tool is a multiparameter water quality measurement probe “EXO2” from EXO (Xylem, White Plains, U.S.A.). This probe was used to measure 6 parameters of a water sample, in order to know the conditions under which the sample was taken. These parameters are the turbidity (in Nephelometric Turbidity Unit - NTU), the depth at which the sample was collected, the dissolved oxygen content (in mg/L), the conductivity (in µS/cm), the temperature (in °C), and the pH. Finally, to localize the site of sample collection, we used a GPS geopositioning system.

## 3. Presentation of the Experimental Area

As the platform and the sensor under development will be used in Amazonian water for the survey of the biochemical quality, we decided to validate our platform in the Lago do Curuaï. This lake is one of the experimental sites on which we carry common experiments with other researchers who ask for new complementary tools for investigations of Amazonian water quality. The Curuaï site (Pará), near Santarém in the northeast of Brazil, has particular characteristics in terms of natural and social dynamics, with biochemical properties related to their filling methods [27,28,29] and with a flooded area ranging between 575 km^2^ and 2090 km^2^ as well as water levels that varied between 3.03 m and 9.61 m between the dry and rainy seasons in one year [28]. The Curuaï site extends approximately 130 km along the Amazon and has approximately thirty interconnected lakes, partly supplied by “Igarapés” of different chemical quality [30]. From the logistical point of view, this site is also interesting because of the large amount of investigation on the water quality of this basin by local [29,30,31] and international [30,32] researchers. Moreover, the proximity of universities and large laboratories working in this area, such as the Insituto Evandro Chagas in the city of Belem (Pará), and the University Federal of Pará, is a useful feature that allows us to compare the results obtained with our system with those of classic analysis tools. Our activity was carried out at the end of June 2019, i.e., at the end of the high-water period.

### 3.1. Several Criteria for Choosing this Experimental Area

**Figure 4:**
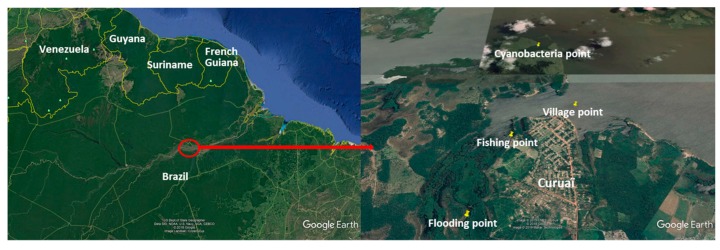
Map of the experimental site and the localization of the collection of the samples (Google Earth image).

To make a survey as effective and relevant as possible with the sensor, we decided to collect water samples at four lake sites in agreement with our colleagues from the University Federal of Pará. These four sites are presented in Figure 4 and classified as a “flooding zone” in an area flooded during the rainy season, a “fishing zone” as used by local residents, a “cyanobacteria zone” as identified by our colleagues from the Federal University of Pará, and a “village zone” close to the village.

### 3.2. Sampling Method and Physical Properties of the Water Samples

For each zone of sampling, samples were collected at three depths in the water column when possible. A specific tool called a Van Dorn sampler was used to collect one liter of water at a specific depth without mixing with some water of another depth. Each sample was stored in a clean plastic container, and the experimental test was carried out within 24 h.

The EXO2 probe was used for a real-time measurement of the water quality of each collected sample during the sampling process. A summary of the physical properties of each water sample measured with the EXO2 probe is presented in Table 1.

## 4. Love Wave Sensor Response

### 4.1. Measurement Principle

The principle of estimating the impact of the liquid is based on monitoring the acoustic sensor response in terms of the gain and phase using the VNA. The portable, open loop readout, electronic setup system presented in Figure 2 mimics the operating principle of the behavior of a VNA over a relatively small range of frequencies. To enable a comparable response between each sample, a baseline of the sensor given by a buffer solution is needed before the injection of the solutions under investigation. In our case, this buffer solution consists of a clean water sample. In aqueous media, for our experiments, we used a strategy of measurement based on a “continuous flow” technique. This method relies on the microfluidic chip presented in Section 2.1.2 and Section 2.1.3. The operating principle for the injection of the liquid consists of using a syringe connected to the outlet of the microfluidic chip by a 0.8 mm/1.6 mm (internal diameter/external diameter) microfluidic tube and a tank containing the sample to test and connected with the same microfluidic tube to the inlet of the microfluidic chip. Thus, by aspiration, the aqueous sample is brought to the sensitive surface of the sensor. This method is chosen to be very simple, avoiding the need for any electrical tool or energy for the circulation of the samples in the sensor. Thus, the first step we carried out before the onsite experiments was to validate our portable platform, compared to the VNA.

**Figure 5:**
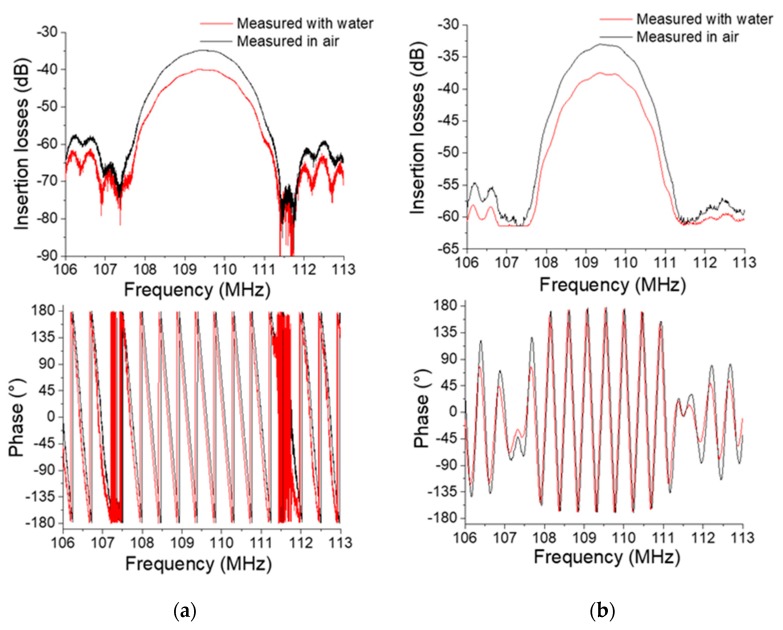
S_21_ frequency domain responses of the Love wave sensor in air and water media measured with the VNA (Vector Network Analyzer) (**a**) and readout electronic module (**b**).

As presented in Figure 5, the response given by the customized readout electronic module shows the S_21_ frequency response comparable to the response obtained by the VNA. Typically, the frequency response of such a Love wave delay line is a bandpass filter with a center frequency of 109.5 MHz and a bandwidth of approximately 4 MHz. More precisely, at ± 1 dB, the absolute values for the maximum insertion losses of the response of the VNA and the module are −34,74 dB and −40 dB in air, respectively, and −33 dB and −37.5 dB in water. Thus, the shift of insertion losses measured between the air medium and the water sample is −5,34 dB measured with the VNA and −4,47 dB measured with the module. Moreover, the relative frequency shift measured at 0° of phase is −40 kHz measured by the VNA and −30 kHz measured by the electronic module.

The results measured by the VNA are higher than those measured by the electronic module. The results appear more precise with the VNA, which is a powerful and expensive tool for radio frequency S parameter measurements, and the results obtained with the electronic module are in good agreement compared to the VNA measurements to validate the behavior of the portable experimental platform for onsite experiments.

### 4.2. An In-field Experimental Test with the Portable Platform

**Figure 6:**
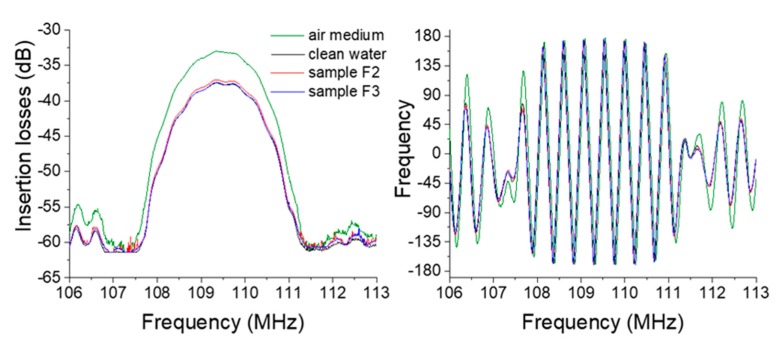
S_21_ frequency domain responses of the Love wave sensor in air and clean water and 2 samples measured with the readout electronic module.

In this part, we present the results obtained in Amazonian water using the proposed experimental portable Love wave acoustic platform. For this goal, we decided to use the operation of the electronic module to determine a fixed frequency and monitor the evolution of both the phase and insertion loss signals versus the time (zero spanning mode) when adding the samples. First of all, all eleven samples were characterized in terms of frequency using the electronic module and the VNA at ambient temperature. As presented in Figure 6, the responses obtained with the electronic module using sensor 1 confirm the ability of the Love wave to propagate with the S_21_ characteristic, which is compatible with a future biochemical detection test.

These typical responses were also obtained with sensors 2 and 3 for each sample of the four areas of investigation. Another set of experiments was also performed using the zero spanning mode with the electronic module. This operating mode is more appropriate for the measurement of biochemical species at low concentrations. Indeed, we obtained a steady state value of 0.03° for the phase, and 0.01 dB for the insertion losses during one minute. This short-term stability is compatible with previously observed signal variation according to the species to detect with the appropriate functionalization [25,33,34].

**Figure 7:**
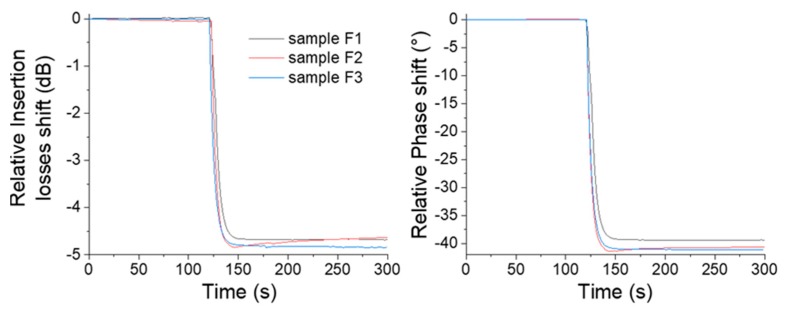
Relative insertion losses and phase time domain responses of the portable Love wave platform for three collected samples.

In this way, Figure 7 presents the response of sensor 1 in real time with a steady-state value in the air medium before the injection of each of the three samples by aspiration of the liquid on the sensor surface. Three sensors were tested and showed similar responses.

### 4.3. Ability to the Love Wave Sensor to Operate in Amazonian Turbid Water

**Figure 8:**
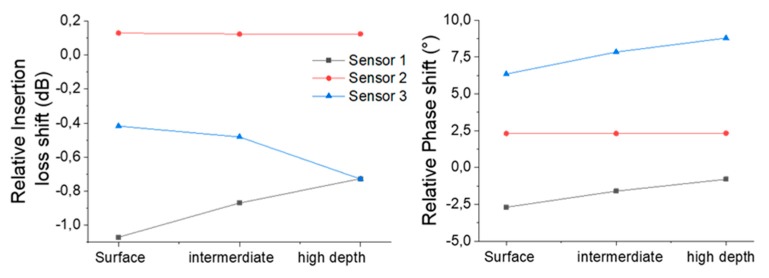
Relative responses (clean water versus samples) of the three sensors at three depths in the cyanobacteria zone.

**Figure 9:**
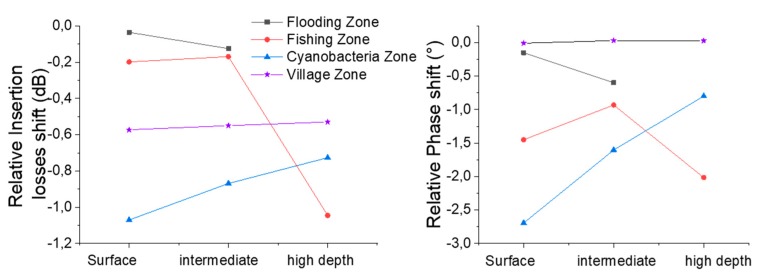
Relative responses (clean water versus samples) of sensor 1 according to the depth for samples in all four zones.

These results allow us to confirm that, first, the electronic module and the sensor are working properly with all the collected samples and, second, that all the samples allow a measurable S_21_ response of the sensor regardless of the physical parameters measured in Table 1. Indeed, the Love wave still propagates without excessive energy losses, which makes the portable platform fully compatible for biochemical detection experiments in field research investigations. Moreover, the ease of transport and use of this platform, when paired with a laptop, allows the realization of in-field processing of the measured data. Figure 8 presents the relative response of the three sensors according to the samples collected at three depths in the cyanobacteria zone, while Figure 9 presents the relative response of sensor 1 for each collected sample at the three depths. This relative response consists of the relative insertion losses and phase measured for a sample relative to the response obtained with clean water.

With this work, and with the results obtained, it would be premature to seek to interpret the curves presented in Figure 8 and Figure 9. Indeed, all these responses are dependent on several physical parameters of the water, which are interrelated and interact with the Love wave. To address this issue, several strategies can be implemented, such as differential measurement in the case of biochemical detection [25,35], a statistical decorrelation approach [36], or physical decorrelation of the experimental response, according to a detailed model of the sensor by taking into account the mechanical and electrical parameters that interact with the propagation of the Love wave.

## 5. Perspectives

The perspectives of this work essentially concern the configuration proposed in this paper with three points involving our current work: the Love wave transducer itself, the electronic module, and the microfluidic chip.

### 5.1. Love Wave Sensor Improvement

To increase the intrinsic sensitivity of and decrease the area occupied by the Love wave transducer, we are now working on an approach that consists of increasing the operating frequency of our delay lines from 109 MHz to 433 MHz. Indeed, as presented in reference [37], this will increase the gravimetric sensitivity of the sensor, provided that the signal-to-noise ratio did not deteriorate. This approach could allow us to obtain a sensitivity of up to four times higher for the 433 MHz delay line while occupying a surface 24 times smaller than that of the 109 MHz device [37]. Moreover, this approach may also more reproducible devices, especially regarding the SiO_2_ guiding layer technological deposition process, due to the decrease in thickness which makes it better controlled with lower rugosity [23]. Technological effects can be further investigated such as those proposed in reference [38]. Moreover, the reduced size of the transducer can allow for a multiple sensor array with the same size as the 109 MHz device. This sensor array could allow for multiple responses, enabling a first decorrelation of the physical parameters that disturb the Love wave propagation.

In the case of biochemical detection, another approach consists of using a sensitive layer that allows an increase in the active surface in contact with the sensing medium by using a porous layer [39]. This strategy, currently under investigation, shows good results for viscous sensing with an increase in the sensitivity of the devices up to a factor of seven due to the relatively high amount of acoustic energy perturbed during the detection mechanism, as demonstrated for viscosity sensing of liquids [39].

### 5.2. Love Wave Sensor Microfluidic Circuit

The accessible sensing surface of the Love wave delay line allows the use of microfluidic chips for the localization of the samples with inlet and outlet orifices. The microfluidic chip we used allows the localization of one aqueous sample on the surface of the sensor. The injection of at least two different liquids without air bubbles and leakage usually requires the use of electrovalves and microsyringes [17]. This strategy is not appropriate for our in-field work, which involves using soft technology processes for the fabrication of the needed components and minimizing the use of electrical energy.

**Figure 10:**
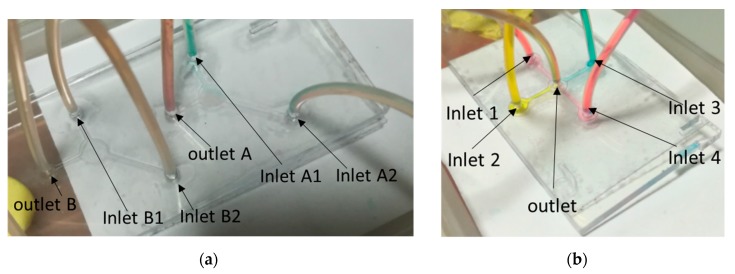
Microfluidic circuits (multiplexor) for injection of multiple samples realized by “xurography”: 2 circuits of 2 inputs to one output (**a**), 1 circuit of 4 inputs to one output (**b**).

A possible solution to this issue consists of using a “xurography” technique for the fabrication of microfluidic circuits [40], by cutting out the microfluidic patterns and combining them with successive layers of materials to create the circuit. It has the advantage of being very inexpensive and requiring few technological resources (clean room, mold, etc.). A first microfluidic circuit using this xurography technique was realized but has not yet been used in the field, as this configuration requires further improvement. Nevertheless, as presented in Figure 10, two microfluidic multiplexer circuits were realized and can be associated with the platform. These configurations of four inlets to one outlet and two inlets to one outlet present very promising results for a platform with a light pretreatment of the water sample.

### 5.3. Electronic Module

The electronic module used in this platform is a very promising tool that can interrogate the sensor in terms of frequency and in terms of “zero spanning” in the field. However, if the performance in terms of the frequency range is lower than the typical performance that we reach with a classical VNA, then this tool fits our needs very well in terms of the frequency response and behavior of our device. More improvement is yet to be undertaken from the hardware and software points of view.

From the hardware point of view, the version we used would benefit from a new version that is able to interrogate two Love wave delay lines simultaneously in transmission by using a switch. This configuration is realized to permit a dual delay line interrogation configuration for differential measurements. The software itself will not undergo significant improvements for these first uses of the device. We plan to adapt the software and the graphical interface to drive the electronic module for the dual delay line configuration, and we also plan to improve the graphical interface to simplify the calibration process, especially for use by a public that lacks experience with the measurement and instrumentation domain.

## 6. Conclusions

This paper presents the results of a in-field experiment with an acoustic Love wave platform. The aim is to validate the ability of such a system to operate in situ with the possibility of extracting useful information from measurements obtained from water samples collected in the field. The relevant properties of this setup including portability, fast implementation, and its use in the field are adapted, and are very interesting for these experimentations. We can conclude that this setup, which consists of a Love wave sensor, an electrical test cell, a microfluidic chip, a readout electronic circuit, and a laptop computer, can provide such flexibility, with interpretable results in the Amazonian Lago do Curuaï floodplain. Second, the impact of the physical parameters of the water does not degrade the acoustic signal of the Love wave and can permit biochemical detection in future work.

Some improvements are now under investigation to, in particular among other tasks, enable a differential measurement with a dual Love wave delay line. This will improve the specificity of the response of the sensor to the target compound in addition to the functionalization of the surface. Moreover, additional work is in progress on the microfluidic circuit, with a special emphasis on making a multiplexor and incorporating a pretreatment of the water sample at low cost before injection onto the sensor.

## Figures and Tables

**Table 1:** Physical properties of the water samples, measured with the EXO2 probe during the sampling process.

Sample Zone	Sample	Sample Depth (m)	Turbidity NTU (Nephelometric Turbidity Unit)	Dissolved Oxygen (mg/L)	Co Nductivity (µS/cm)	pH	Tempera-Ture (°C)
Flooding	W1	0.958	6.9	1.29	10.7	5.22	27.72
	W2	0.219	6.9	3.94	19.4	5.95	29.95
Fishing	F1	4.9	3.92	0.58	16.3	5.65	27.89
	F2	2.375	1	4.76	33.2	6.55	30.11
	F3	0.127	1.85	6.18	34.3	6.78	30.64
Cyano-bacteria	C1	3.544	3.90	2.39	39.7	6.40	29.74
	C2	1.544	5.62	3.69	40.0	6.50	30.20
	C3	0.185	5.66	3.85	40.1	6.50	30.28
Village	V1	3.112	4.05	6.14	36.6	6.77	30.21
	V2	1.568	4.20	6.21	36.5	6.79	30.24
	V3	0.205	4.12	6.24	36.5	6.79	30.26
